# A novel lineage of large aquatic bacteriophages identified through metagenomics

**DOI:** 10.1007/s00705-025-06455-7

**Published:** 2025-11-15

**Authors:** Carolina Perez-Hernandez, Joud Aldaroub, Zachary K. Barth, Frank O. Aylward

**Affiliations:** Department of Biological Sciences and Center for Emerging, Zoonotic, and Arthropod-borne Pathogens, Virginia Tech, 926 West Campus Drive, Blacksburg, VA USA

## Abstract

“Jumbo phages” are tailed phages with genome sizes >200 kbp and physical dimensions reaching up to 0.45 μm. Although jumbo phages represent only a small fraction of the isolated phages to date, metagenomic surveys have shown that they are broadly distributed in a wide range of environments. In this study, we surveyed metagenomic data from aquatic systems and identified 25 genomes from a heretofore-undescribed lineage of jumbo phages with genomes reaching up to 307 kbp. We refer to these phages as “moraphages”, from the Gaelic word ‘mór’, for large. Moraphages represent a diverse lineage with inter-genome average amino acid identity (AAI) ranging from 39 to 95%, and our pan-genomic analysis identified only 26 viral orthologous groups (VOGs) found in at least 80% of the genomes. Our phylogenomic analysis suggests that moraphages are distant relatives of a recently described lineage of huge phages from marine sediment. Moraphages lack much of the genetic machinery found in other lineages of large phages, but they have a range of genes that may be used to take over host cellular machinery and subvert host defenses, such as glutamine synthetases, antitoxin genes, and chaperones. The predicted hosts of most moraphages are members of the phylum *Bacteroidota*, and some encode homologs of the chaperones DnaK and DnaJ that bear evidence of recent gene transfer from members of the order *Flavobacteriales*. Our work sheds light on the emerging diversity of large phages that are found across the biosphere.

## Introduction

Bacteriophages, also called phages, are viruses that infect bacteria and are found in diverse environments. They are the most abundant and diverse biological entities on the planet, with estimates of 10^31^ phage particles existing on Earth [1]. Phages modulate the abundance and activities of microorganisms that make up a large component of the biomass on Earth [2] and have been used to advance our understanding of molecular, environmental, and evolutionary biology [3]. Phages have been isolated from soil, marine environments [4], wastewater [5], and even acidic hot springs [6], amongst other ecosystems, and have adapted to be the ever-present predators of bacteria. Phages are a source of enormous genetic diversity but have gone relatively unnoticed when compared to other biological entities. Lytic phages control the population size of the bacterial host [7] and release valuable nutrients from within bacterial biomass into the environment that organisms at other trophic levels can then access [8, 9]. Obligate lytic phages are a potential tool that can be harnessed to combat the rising number of infections caused by bacteria that are resistant to commonly used antibiotics. This property and its applications extend to other realms as well, including biocontrol in agriculture and wastewater treatment.

Among the many phages that exist across all environments, those that have particularly large physical dimensions and genome lengths have remained largely unknown until recently. Referred to as “jumbo phages,” these predominantly lytic tailed phages have a genome size >200 kbp and have physical dimensions near 0.5 μm [10]. Isolation of large phages poses a number of challenges when using conventional methods. Somewhat counterintuitively, jumbo phages tend to form small plaques and are often overlooked in favor of more visible and easily isolated plaques. Plaque assays using higher percentages of agar prevent jumbo phage virions from diffusing through the agar matrix and forming visible plaques on the bacterial overlay [11]. Filters are commonly employed when preparing samples and may contribute to the bias against their detection and isolation. While removing bacteria and other organisms is necessary for all phage isolation, the use of filters may not be the optimal approach for jumbo phages [12].

When comparing jumbo phages to their small-genome counterparts, the makeup of their genome is quite different. Some jumbo phage lineages have been shown to have decreased dependence on host cellular machinery and often encode their own genes for genome replication and gene expression, including DNA polymerases and multi-subunit RNA polymerases (RNAPs) [13, 14]. There is a correlation between the presence of tRNAs and genome size, with the number of tRNAs encoded increasing as a genome increases in length [12]. The presence of tRNA genes in jumbo phage genomes might have arisen due to the host’s own tRNA molecules becoming compromised during phage infection [15]. Other genes unique to jumbo phages include proteins that circumvent the bacterial CRISPR-Cas defense system. Certain jumbo phages have been found to form a selectively permeable “phage nucleus” within the bacterial cytosol, a structural feature thought to be associated with eukaryotic evolution [16]. This nucleus-like compartment acts as a physical shield that protects the phage genome from bacterial defenses when replication is underway. Some investigators have postulated that jumbo phages have a broader host range due to genes that reduce dependence on the host, allowing for efficient virion and genome production in many different strains or across species [17–19].

Due to the challenges of isolating jumbo phages from environmental samples, recent studies have used metagenomic methods for identifying jumbo phages. This approach is also challenging due to the difficulty of resolving large genomes, but continual advances in assembly algorithms over the last few decades have improved our ability to recover large phage genomes, and several approaches have been developed to make use of long-read sequencing, bin phage contigs, or iteratively resolve large scaffolds [20–22]. Metagenomic approaches have led to a dramatic increase in the number of complete jumbo phage genome sequences determined in recent years and have revealed some of the largest phage genomes ever identified [20, 23–26]. In this study, we sought to identify novel lineages of jumbo phages by mining metagenomic data, primarily from freshwater systems. We used a recently proposed phylogenomic framework for phage diversity [27] to demarcate a new group of 25 phage genomes. We present a phylogenomic analysis of these phages and discuss the notable genes encoded in their genomes.

## Methods

To search for large phage genomes across a range of different habitats, we surveyed 16,801 metagenomes from the SPIRE database [28]. We chose the metagenomes in this set to represent a wide range of environments, including freshwater ponds and lakes, marine habitats, soil and sediment samples, engineered systems such as wastewater treatment plants, and others. For this study, we downloaded assemblies that were already available in the SPIRE database and included only contigs >200 kbp in length in the analysis. These contigs were analyzed using geNomad v 1.8.0 with default parameters, and only contigs that were classified as belonging to members of the class *Caudoviricetes* were selected.

Large phage contigs were screened by iteratively constructing trees based on VOG profiles using VirTree [27] and visualizing the output in iTOL [29]. In these trees, we included a reference set of phages that was previously constructed from the Inphared database and various metagenomic datasets [27, 30]. While examining trees, we sought to find clades of phages with large genomes that did not include cultivated strains. This resulted in a set of 25 SPIRE contigs that formed a distinct clade with high bootstrap support and formed a divergent group with no obviously cultivated representatives. These contigs ranged in length from 200,148 to 306,874 base pairs, and geNomad analysis showed that four had direct terminal repeats (DTRs) indicative of complete phage genomes. The closest relatives of these phages in our database were three “ERM phages” that were previously reported in a metagenomic survey of large bacteriophages [26], and we therefore included these in our subsequent analyses as well. Because these phages represent a novel clade that, to our knowledge, has not been described previously in the literature, we will refer to them here as “moraphages”, from the Gaelic word ‘mór’, for large.

### Phylogenetic trees of marker genes and average amino acid identity

We constructed phylogenies of the moraphages together with divergent relatives, using three viral marker genes: the major capsid protein (MCP: VOG00292), terminase large subunit (TerL: VOG00012), and family B DNA polymerase (PolB: VOG00275). For each of these trees, reference sequences in the NCBI database were identified using BLASTp, with representative moraphage proteins as the query. Amino acid sequences were aligned using Clustal Omega with default parameters [31], and trees were constructed using IQ-TREE multicore v. 2.3.4 [32, 33]. The.treefile file of the output was visualized in iTOL [23]. This same approach was used to make trees of the viral DnaK homologs identified in some moraphage genomes (see below). Average amino acid identity was calculated as described previously [34, 35], and figures were made using the pheatmap function in R.

### Protein prediction and functional annotation

Proteins were predicted using Prodigal v. 2.6.3 with default parameters [36]. Functional annotation was done using the eggNOG-mapper v2 (emapper-2.1.12) [37] tool based on eggNOG v. 5.0 [38]. In addition, we compared all of the proteins to the VOG database v. 214, using hmmsearch in HMMER3 (e-value cutoff, 1e-5 [39]).

### Orthogroup analysis

We used OrthoFinder v. 2.5.4 [40] to identify orthologous groups (OGs) in the moraphages (-og parameter). We selected an 80% cutoff, meaning that the core genome would consist of the OGs that are shared with 80% or more of the phages. The three ERM phages were counted as one occurrence, and this percentage was therefore calculated out of 26 rather than 28 phages. The core genome was identified based on occurrence rather than the number of hits to an OG a phage may have. The predicted functions of the core genome OGs were predicted manually using BLASTp and HHpred. From the OrthoFinder results, which compiled amino acid sequences into their respective OGs, sequences were input into each database, and the best annotation was chosen based on percent identity and e-values. Pan-genomic statistics were compiled using the micropan utility in R.

### Host prediction based on homology search

The predicted proteins encoded by each phage genome were searched against the RefSeq v. 227 database using LAST (parameters -m 500), and the best hits were recorded. The phylum of each best match was identified, and the bacterial phylum *Bacteroidota* received the most best hits for almost every phage. To assess the confidence that members of the *Bacteroidota* were the hosts of the phages, we calculated a metric by dividing the number of best hits to the *Bacteroidota* by the number of hits to the next-most-prevalent phylum and expressed this value as a ratio. If the ratio exceeded 1.5, the most prevalent host phylum was chosen as the likely host.

### Synteny plot

A synteny plot of the complete phage genomes was made using GenoPlotR [41] in the R programming environment. The genomes and gene predictions were used as input, together with pairwise BLASTp outputs (BLASTp version 2.16.0+, e-value threshold of 1e-10 [42]).

## Results and discussion

### Phylogenetic placement of the moraphages

Here, we examined a novel clade of large phages found in metagenomes generated from a wide range of environments. The genome length of the moraphages ranged in size from 200 to 307 kbp, and four contained DTRs indicative of potentially complete genomes. Moraphages were identified primarily from freshwater sources such as lakes and rivers, but some variety in their environment was found (Table 1). For example, moraphages were also found in human-made facilities, as one phage was obtained from reclaimed water used to irrigate crops, and another was obtained from sediment near a natural gas well. Three distinct natural environments apart from freshwater are also represented: a marine water column, a coastal salt marsh, and an estuary. Out of the four moraphages with complete sequences, three were of freshwater origin, and one was isolated from an estuary. Throughout our genomic and phylogenetic analyses, we observed that the moraphages are related to three “ERM phages” that were recently discovered in marine sediment metagenomes [26]. We therefore also included these genome sequences in most of our comparative analyses. Other reference phages that were included in our phylogenetic analysis were found by comparing moraphage marker genes to entries in the NCBI databases, which showed similarities to smaller phages derived from various sources, including aquatic systems as well as the human gut and mouth.

**Table 1:** Moraphages and their environmental origins

**Phage ID**	**Environment**
SAMN06267920_k141_159460.fasta	Freshwater
SAMN12796234_k141_1275679.fasta	Freshwater
SAMEA8660007_k141_1141498.fasta	Freshwater
SAMEA6649935_k141_278539.fasta	Freshwater
SAMEA6265476_k141_1882880.fasta	Freshwater
SAMN11356859_k141_1778059.fasta	Freshwater
SAMEA6265477_k141_410594.fasta	Freshwater
SAMEA8660000_k141_1707698.fasta	Freshwater
SAMN12794754_k141_102644.fasta	Freshwater
SAMEA6380161_k141_158953.fasta	Freshwater
SAMEA6380159_k141_2950544.fasta	Freshwater
SAMN10386151_k119_19505.fasta	Freshwater
SAMN08331091_k141_1934352.fasta	Freshwater
SAMN10386151_k119_131298.fasta	Freshwater
SAMN10386137_k119_135130.fasta	Freshwater
SAMN11532505_k141_984480.fasta	Freshwater
SAMN11532503_k141_1797313.fasta	Freshwater
SAMN11533406_k141_1557757.fasta	Freshwater
SAMN11530718_k141_1870712.fasta	Freshwater
SAMN11532462_k141_687100.fasta	Freshwater
SAMN10386142_k119_302602.fasta	Reclaimed water/recycled water
SAMN19228552_k141_217681.fasta	Estuary
SAMN09092241_k141_1008493.fasta	Coastal salt marsh
SAMN08778078_k141_2211054.fasta	Marine water column
SAMN10350962_k141_248555.fasta	Subsurface sediment (gas well)
ERM_PHAGE_CIR_34_117.fasta	Marine sediment
ERM_PHAGE_CIR_34_14.fasta	Marine sediment
ERM_PHAGE_CIR_75_34.fasta	Marine sediment

**Fig. 1:**
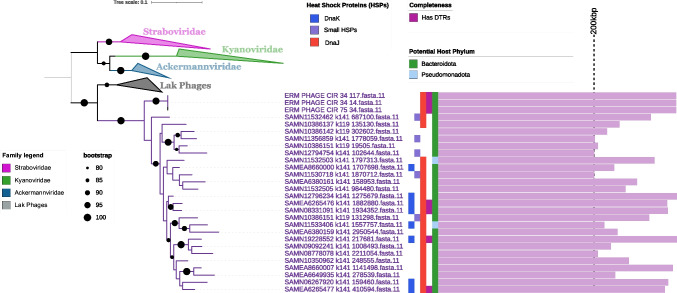
Phylogenetic tree with host phylum prediction. The reference phages used in this tree are members of the T4 group and Lak phages, a group that includes phages with some of the largest genome sizes ever recorded. In this tree, the moraphages clustered with each other. Interestingly, the group 1 and Lak phages can be seen to share a more recent common ancestor than the one shared with the T4 phages. Their hosts are predicted to belong to the same phylum – *Bacteroidota* – with the host of the Lak phages being narrowed down to members of the genus *Prevotella*. The presence of heat shock genes in all of the moraphage genes is also indicated in the figure, as it was the basis for host prediction

The moraphage sequences were compared with those of large phages of other lineages using VirTree to determine whether they share similarities with members of these groups (Fig. 1). Also included in this analysis were members of the T4 group, specifically the families *Straboviridae, Kyanoviridae*, and *Ackermannviridae,* as well as the Lak phages, which have been found in human and animal gut metagenomes [23, 43]. The resulting phylogeny showed that moraphages are a distinct lineage that does not fall within any of these established groups of large phages.

To identify the possible hosts of moraphages, we performed a homology search of all proteins in the phage genomes against the NCBI RefSeq database to identify hits to host bacterial lineages as described previously [26]. This analysis was performed on the grounds that phages often acquire genes from their host and that homology searches often provide clues regarding the identity of the host. From this analysis, we determined that the likely hosts of most moraphages are members of the class *Flavobacteriia*, with the plurality of best hits for 23 phages falling within the phylum *Bacteroidota*, while the plurality of hits for two were to members of the phylum *Pseudomonadota* (Fig. 1). The prediction of a host within the *Bacteroidota* is also consistent with phylogenetic analysis of the heat shock proteins identified in the moraphage genomes (see details below).

**Fig. 2:**
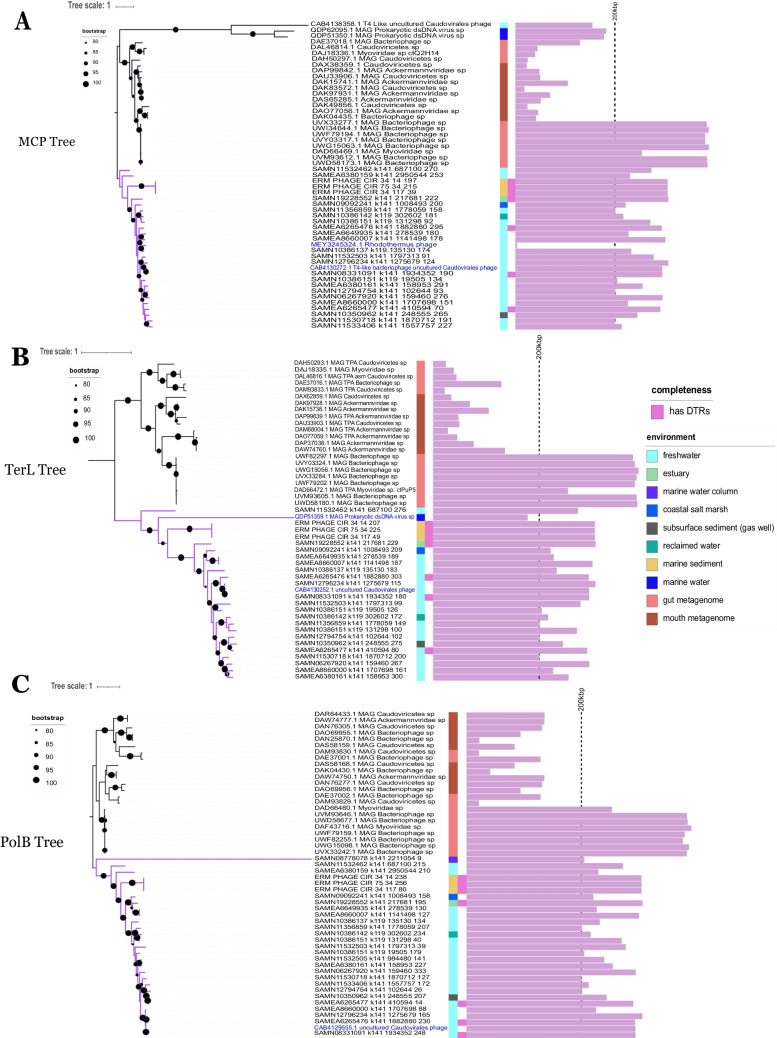
Phylogenetic trees based on MCP, PolB, and TerL sequences. The group 1 phages formed a clade with each other (purple branches) based on shared VOGs in all trees. Most of the reference phages also formed a clade (black branches). The majority of the moraphages were collected from freshwater environments, but other environments such as marine water and sediments are represented. The three ERM phage sequences and four of the 25 moraphage sequences are considered complete due to the identification of DTRs. Bootstrap values range from 80 to 100 across the tree. (**A**) One *Caudovirales* phage and one phage infecting *Rhodothermus* (both labeled in blue) were not included in the group 1 clade, and both are of freshwater origin. The genome size of the *Rhodothermus* phage is small because the sequence is incomplete, but within this limited genome source, a match for the MCP was identified. (**B**) The TerL tree includes a *Caudovirales* jumbo phage and a MAG prokaryotic dsDNA virus (both labeled in blue) that clustered with group 1. The prokaryotic dsDNA virus is not a jumbo phage but does approach the 200 kb benchmark and is of marine origin. (**C**) A single reference jumbo *Caudovirales* phage (labeled in blue) was found to be closely related to the moraphages of freshwater origin. The longest branch on this tree belongs to one of the moraphages, suggesting that its PolB is more divergent from those of the other phages. This phage is also the only one that is of marine water column origin

To determine the phylogenetic placement of this novel clade of jumbo phages relative to other phages, phylogenetic trees were constructed based on the terminase large subunit (TerL: VOG00012), major capsid protein (MCP: VOG00292), and family B DNA polymerase (PolB: VOG00275) sequences. When constructing these trees, we included a set of the best BLASTp hits to these proteins (Fig. 2, panels A-C). Some of the moraphage sequences did not show any similarity to MCP, TerL, or PolB VOGs and were therefore excluded from further analysis. In all of these trees, the moraphages formed a distinct clade that was divergent from any reference strains found in the NCBI database, confirming that they form a unique lineage of large phages. It should be noted that not all of the reference phages were jumbo phages, despite encoding proteins with sequence similarity to jumbo phage proteins. A common trend can be seen in all three trees, as the majority of the reference phages did not form a clade with the moraphages, resulting in two major nodes. A second trend was observed regarding the environments of the phages included in each tree. The reference phages, with a few exceptions, had been identified in metagenomic samples from the human gut or mouth. The trees suggest that the moraphages are a diverse lineage of phages that are distinct from previously characterized groups, and we therefore sought to quantify the genomic diversity within this lineage.

**Fig. 3:**
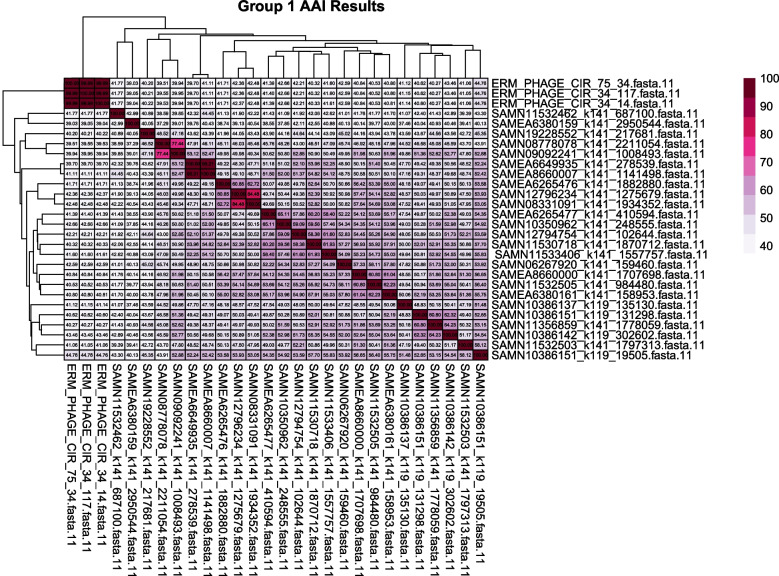
Heat map displaying the amino acid identity between the members of group 1. The amino acid sequences of all group 1 phages were compared with each other using the AAI index. The majority of the phages have low identity values, ranging from 39% to 65%. The ERM phages are essentially identical in their sequences and have the highest values assigned to them (100). Three pairs of phages were above the 65% threshold, with values of 77.44%, 84.48%, and 95.21%, respectively. The pair with the highest sequence similarity were from freshwater sources, and the pair with the third highest similarity were of coastal salt marsh and marine water column origin

We also compared the moraphages to each other by examining their pairwise average amino acid identity (AAI), which is an index used to quantify whole-genome relatedness. We visualized these results using a heat map and a clustering dendrogram (Fig. 3). The values are mostly in the low percent identity range, with the majority of phages falling between 39 and 65%, suggesting that this lineage has broad diversity. There are some clear outliers from this average, namely three pairs of phages that have a high degree of amino acid sequence similarity. The pair with the second-highest identity value of 84.48% (SAMN12796234:SAMN08331091) and the pair with the highest identity value of 95.21% (SAME6649935:SAME8660007) originated from freshwater samples. Interestingly, the pair with the third-highest identity value, at 77.44% (SAMN09092241: SAMN08778078), are from different environments, from a coastal sea marsh and a marine water column, respectively. The phage SAMN09092241 contains a PolB gene but appears to lack an MCP or TerL VOG gene and, this phage was judged to have the most divergent PolB due to the length of its branch (Fig. 2 A-C). In sharp contrast, its paired phage SAMN08778078 had hits to all three hallmark proteins. The pair with the highest identity value were isolated from different freshwater locations but clustered together in all three trees. However, it should also be noted that the only phage genome out of these pairs that has DTRs was that of SAMN0833109, suggesting that, despite these phages being highly similar to each other, the AAI values of these three pairs may have been based on AAI measurements of incomplete genome comparisons.

### Pan-genomics of the moraphages

**Fig. 4:**
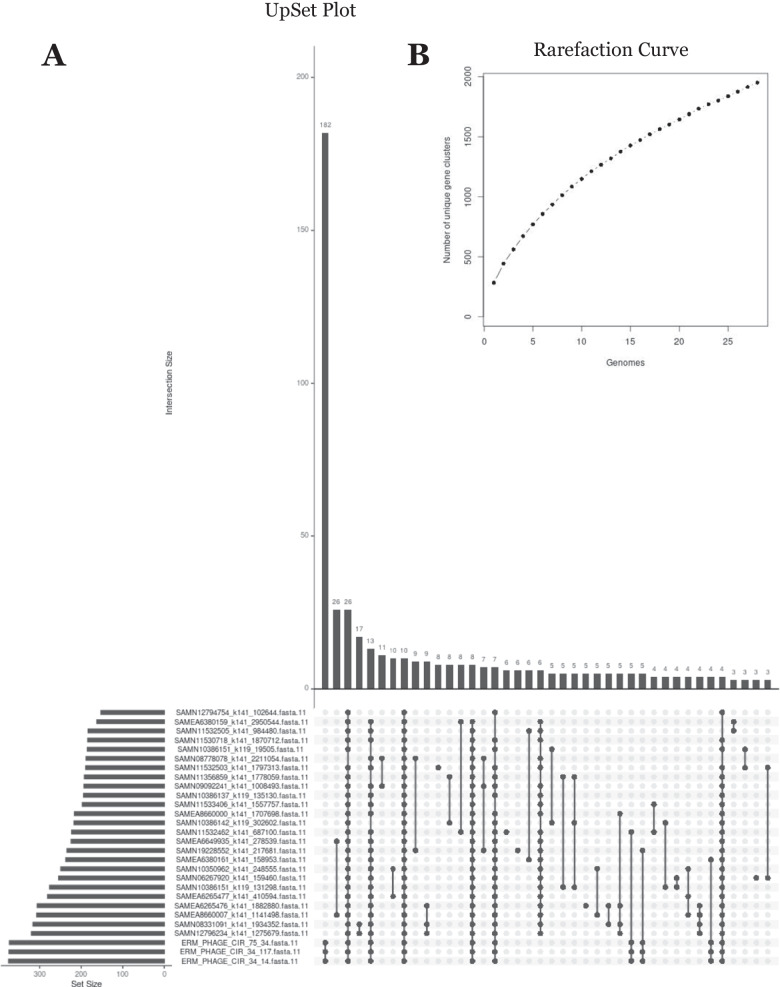
UpSet plot and rarefaction curve generated from the pan-genomic analysis. Using the amino acid fasta files of each phage in group 1, a pangenomic analysis was conducted using OrthoFinder. The orthogroups generated were used to determine the core genome of the moraphages. (**A**) The ERM phages are an outlier when comparing them to each other, as they share many of the same genes, indicating that their genomes are essentially identical. The core genome consists of a relatively small subset of genes, and this can be attributed to the increasing number of unique gene clusters that appear as more genomes are added to the pan-genomic analysis, as seen in the rarefaction curve in panel **B**

The majority of the moraphages have low AAI percentages to each other, and we investigated if there were any genes that they shared as a group by assembling a pan-genome. The core genome consists of genes that are shared between all or the majority of the members of the clade, which offers a more focused view into the proteins that define the phage lineage. The pan-genome analysis also assesses the degree of gene diversity within the moraphage genomes by plotting a rarefaction curve (Fig. 4). While the moraphages may share a set of essential genes, the majority of their DNA encodes divergent and novel genes.

As all three ERM phages shared the same OGs, they were counted only once rather than three times for the core genome analysis. Many phages also had multiple hits to the same OG, but we identified the core genome based on occurrence. Our analysis showed that there are only 10 OGs that are shared between all moraphages, while 98 OGs are shared between 80% of them or more. To gain insight into the function of these core OGs, we manually investigated their predicted functions using BLASTp and HHpred (see Methods). There are some notable results from both BLASTp and HHpred analysis, namely that four separate OGs (0000011, 0000025, 0000026, and 0000028) are putative glycosyltransferases. The function of these proteins in phages has been reported to be glucosylation of phage DNA, which protects it from host restriction endonucleases as part of the phage replisome. Glycosyltransferases have also been observed in temperate phages, modifying host serotypes during lysogeny [44]. Genetic material is not the only target of glycosylation, the virion itself can be altered as well. Glycans have been observed at the C-termini of both the capsid and tail tube subunit of *Mycobacterium* phages, acting as a shield against anti-phage responses [45]. Due to their diverse roles, the acquisition of diverse glycosyltransferases has occurred many times independently in different phage lineages [46], and there is great diversity in their structure and function. Apart from this, structural proteins such as baseplate proteins, contractile tail proteins, and capsid assembly proteins also make up the core genome, which can be expected, as all of these components are necessary for the assembly of a virion. However, a gene encoding a tape measure protein (TMP), an essential tail assembly protein, was not identified within the genomes. The length of the TMP gene has been shown to be correlated with the length of the phage tail [47], but it might also have other important functions, such as peptidoglycan-degrading activity [48]. As phage tails are used for infection of the host and injection of the phage genome, the TMP is believed to contribute to this process [49]. Because of its importance for large tailed phages, we believe that the moraphages have a TMP gene within their genome that was not detected because of its divergence from its homologs in other phages.

Also of note, OG0000024 and OG0000031 are associated with RNA polymerase, serving as a sigma factor and a mediator of transcription, respectively, and OG0000043 was identified as a polymerase B subunit (see Data availability for raw data files). These results indicate that proteins associated with DNA replication, transcription, and virion structure are the most prevalent of the shared genes in the moraphage core genome. We also examined a set of 26 VOGs that were found in >80% of the moraphage genomes, which once again includes genes for a range of protein families predicted to be involved in DNA replication, transcription, and virion structure (Table 2).

**Table 2:** VOGs shared by moraphages

**VOG**	**Occurrence**	**Annotation**
VOG00031	24	DNA primase
VOG00042	24	Exonuclease subunit 2
VOG00043	23	Glutaredoxin
VOG00048	27	Deoxyuridine 5'-triphosphate nucleotidohydrolase
VOG00075	27	Tail tube protein gp19
VOG00084	27	Baseplate wedge protein gp25
VOG00090	24	RNA polymerase sigma-F factor
VOG00112	24	Terminase, large subunit
VOG00127	27	Tail sheath protein
VOG00275	27	DNA-directed DNA polymerase
VOG00292	26	Major capsid protein
VOG00369	27	Neck protein gp13
VOG00439	24	Lysine-tRNA ligase
VOG00674	25	Prohead core protein protease
VOG00681	25	Portal protein
VOG00710	25	DNA-directed DNA polymerase
VOG01084	27	Baseplate wedge protein gp6
VOG01986	28	DNA polymerase I
VOG02072	24	ATP-dependent Clp protease ATP-binding subunit ClpA
VOG02083	24	Hypothetical protein
VOG02246	23	Single-stranded DNA-binding protein
VOG02439	27	PhoH-like protein
VOG05220	24	Hypothetical protein
VOG06156	27	Hypothetical protein
VOG17084	25	ATP-dependent DNA helicase uvsW
VOG18190	24	Putative peptidase

**Fig. 5:**
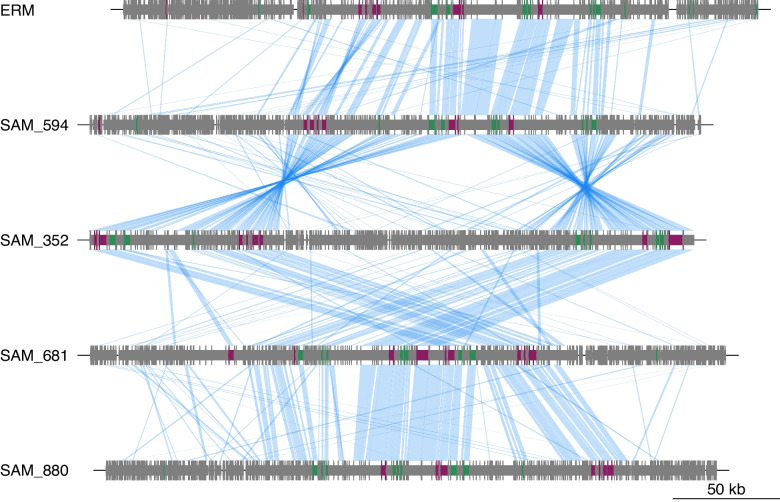
Synteny plot of the complete genomes of four moraphages and one representative ERM phage. Blue lines denote the best BLASTp matches of the encoded proteins. Genes colored in green are those with matches to VOG families involved in DNA replication (polymerases, helicases, primases, etc.), while those colored in maroon are those with hits to VOG families involved in virion morphogenesis (major capsid proteins, terminase subunits, tail fibers, etc.). The names are abbreviated for clarity: ERM, ERM_PHAGE_CIR_34_14; SAM_594, SAMEA6265477_k141_410594; SAM_352, SAMN08331091_k141_1934352; SAM_681, SAMN19228552_k141_217681; SAM_880, SAMEA6265476_k141_1882880

We also constructed a synteny plot to visualize genome-level variability between the moraphages (Fig. 5). For this, we used the four moraphages that were predicted to be complete based on the presence of DTRs, as well as one representative ERM phage. This analysis confirmed the presence of conserved blocks of homologous proteins encoded by the phages, which in many cases correspond to genes involved in virion morphogenesis and genome replication. Moreover, it is apparent that several inversions and other genomic rearrangements have taken place in the evolution of this group, suggesting that these may be important events that shape their genomic organization. Lastly, the synteny plots revealed several genomic regions with no or little homology to related phages, suggesting that they may represent genomic islands with variable composition across this lineage. If this is correct, an important avenue of future research would be to examine the functional repertoires of these islands and the dynamics of their gain and loss among the moraphages.

### AMGs and other functional genes encoded by the moraphages

While the moraphages encode many unique proteins (discussed below), there are many that are missing that have been identified within other large phage lineages. For example, in a study by Al-Shayeb et al. [26], some clades of jumbo phage from many environments were found to encode tRNA synthetases that are distinct from those of their host, but we did not find any of these in the moraphage genomes. Another significant discovery was the “phage nucleus” that forms in the host during infection by phages of the family *Chimalliviridae* [16, 50]. Members of this family were found to encode a multi-subunit RNA polymerase for the transcription of viral genes within the “phage nucleus”, limiting the phage’s dependence on the host. We did not find any RNA polymerase gene homologs in the moraphages, suggesting that this clade of viruses is dependent on host transcriptional machinery for gene expression. This also suggests that the moraphages have a distinct infection program that differs from those of other lineages of large phages that have been characterized previously.

Of the 25 novel moraphage sequences, only four contained DTRs and are therefore presumed to represent complete genomes. We therefore performed detailed functional analysis using these four genome sequences. For this, we performed manual curation of protein annotations using BLASTp and HHpred, as well as cross-referencing with our automated annotations using EggNOG (see Methods). Using this approach, we found that these four moraphages were predicted to encode a histone H1-like protein (PF07432). Also referred to as a “linker histone”, this protein helps to stabilize the nucleosome by linking adjacent nucleosomes together [51], and homologs have been identified in the genomes of giant eukaryotic viruses such as medusavirus [52]. Medusavirus has been shown to encode a full set of histone homologs, but this H1-like protein is distinct in the timing of its expression. In contrast to the other histones, H1 is expressed early in the infection cycle and may perform independent functions such as shutting down the host’s genome or regulating the virus’s genome for transcription [53]. A homolog of H1 has also been identified in *Chlamydia trachomatis* as Hc1, which binds to and densely compacts chromatin, regulating chlamydial gene expression [54, 55]. Hc1 has been expressed in *E. coli* to observe its effect on DNA structure and transcription/translation, and it was found that protein and DNA synthesis in the Hc1-expressing strain was considerably reduced [56, 57] Although the function of these histone-like proteins in moraphages is still unknown, it is intriguing to consider that the phage may use it to compact its own genome during packaging in a manner analogous to that described for giant viruses [58, 59].

Looking at the moraphages as a group, nine of them encode AMG glutamine synthetase (GS), whose function in bacteria is associated with nitrogen metabolism and ammonium reassimilation [60, 61]. This protein has been identified in oceanic viruses, suggesting that these viruses may manipulate nitrogen cycling in their environment [62]. GS components can also be used to activate alternative nitrogen-producing pathways under nitrogen-starved conditions [63]. Since the moraphages are of aquatic origin, nitrogen usage and potentially limited access to this element may have contributed to the phage’s acquisition of a GS,

We also identified multiple antitoxin proteins in the four genomes with complete sequences. Antitoxins are typically components of toxin-antitoxin (TA) systems and are a common genetic module found in both bacterial and archaeal chromosomes and plasmids, with some species encoding multiple distinct systems [64]. TA systems have also been commonly identified in prophage genomes [65, 66]. These modules typically comprise a growth-inhibiting toxin and its antitoxin, allowing for reversible changes to the bacterial cell, including some in response to phage infection [67]. Recently, there has been renewed interest in the anti-phage activity of TA systems. Bacteria use the modules as a broad method of defense against phage infection, with various biochemical activities that can target different phage processes [68]. Some phages are known to use antitoxin mimics to abrogate restriction by anti-phage TA systems. This has led to speculation that freestanding antitoxins in phage genomes may act as a counter to host anti-phage immune systems. The abundance of antitoxin genes in moraphage genomes may reflect an investment in countering host cell defenses.

Intriguingly, we observed an association between CcdA/HicB-like antitoxins and a group of Pac-I-like putative homing endonucleases present in multiple copies in some of the moraphage genomes. Homing endonucleases are selfish genetic elements that can spread within phage genomes and facilitate allelic conversion during phage coinfection [69]. In addition to neutralizing toxin activity, CcdA and HicB are known to transcriptionally repress toxin expression [70]. The T4-encoded homing endonuclease Itev-I has been shown to repress its own promoter [71]. Pairing an antitoxin gene with a homing endonuclease in a single module may be an alternative strategy for regulating homing endonuclease activity. Alternatively, if the antitoxin provides a fitness benefit to the phage, pairing this gene with a homing endonuclease may be a way to offset any fitness cost imposed by the selfish endonuclease.

One of the most striking features of the moraphages is the presence of several heat shock proteins (HSPs), molecular chaperones that are widespread in plants, animals, and prokaryotes, regulating the folding of proteins and protection from improper folding [72]. Similar to antitoxin mimics, phage-encoded HSPs may be a means of overcoming anti-phage defenses. HSPs are known to promote antiviral responses in eukaryotes [73], and recent work has shown that DnaJ is required for the anti-phage activity of an NLR-like protein in *E. coli* [74]. Additionally, another bacterial chaperone, SecB, is required for the function of some antitoxins [75], and some HSPs are able to phenocopy SecB’s canonical function for protein secretion [76]. As an alternative to a counterdefense function, phage-encoded DnaJ chaperones might recruit the host’s DnaK and facilitate the assembly of viral proteins, as seen in eukaryotic viruses such as simian virus 40 [77] and crocodilepox virus [78]. In a 2012 study, a T4-related enterobacteriophage named RB43 was found to encode a putative functional J-domain protein with 63% amino acid sequence identity to known *Escherichia coli* DnaJs [79]. DnaJs also seem to be more common in the moraphage genomes, as can be seen in Figure 1. While these proteins are highly conserved across the domains of life, their presence in phage genomes and their overall role in phages is not well understood.

In addition to DnaJ and DnaK, some moraphages encode the small heat shock protein HSP20. A subclass of HSPs, small heat shock proteins (sHSPs), with a few exceptions, are also ubiquitous in all forms of life but differ from HSPs. These proteins have a low molecular weight, ranging from 16 kDa to 42 kDa, and exhibit lower nucleotide and amino acid sequence conservation across species groups [80, 81]. Like other HSPs, these proteins have been found to participate in host protein folding and mediated disaggregation of proteins [82] and to respond to environmental changes [83]. The number of sHSPs in different organisms is also quite variable, with humans having 10 [84], *Drosophila* having four [85, 86], and some pathogenic bacteria such as *Helicobacter pylori* not possessing any [87].

**Fig. 6:**
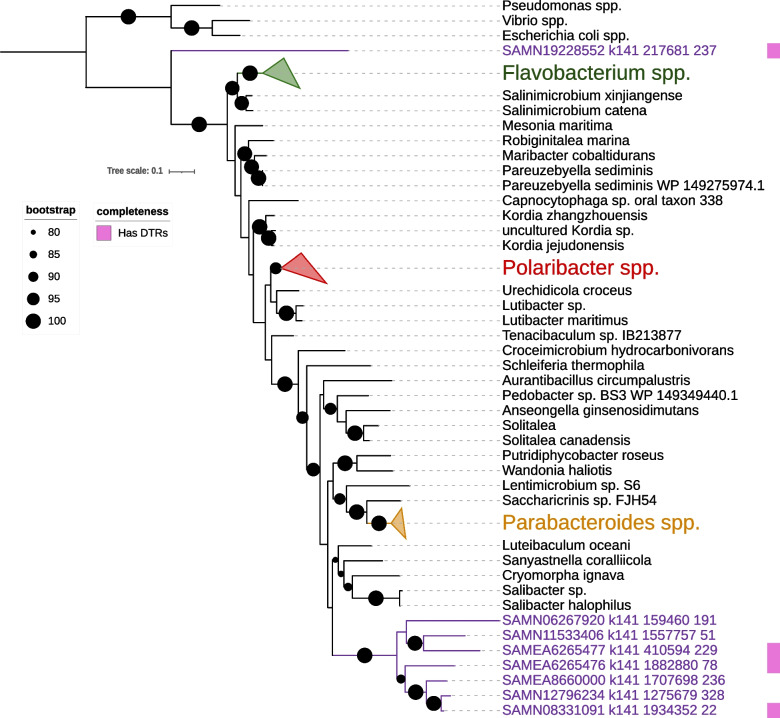
DnaK tree with members of the phylum *Bacteroidota* as references. All moraphages that encode a DnaK protein were included, together with representative best BLASTp hits of these proteins in the NCBI RefSeq database (searches performed in May 2025). All but one of the phage DnaKs formed a clade within the *Bacteroidota* lineage. Phage SAMN19228552 was the only exception; the DnaK homolog from this phage was highly divergent and might have been acquired independently

Using HSP70 (DnaK) sequences from the eight moraphages encoding the protein and related sequences from bacteria indentified by BLASTp searches of the NCBI RefSeq database, a phylogenetic tree was constructed. One particular moraphage (SAMN19228552) displayed a relatively long branch length and did not cluster with the other seven DnaK-encoding moraphages, suggesting that it might have acquired a Dnak gene independently from the others. The other seven moraphage DnaK homologs formed a distinct clade within the family *Flavobacteriaceae* of the phylum *Bacteroidota*, consistent with the view that these phages likely infect these hosts and acquired HSP70 from a member of this bacterial lineage (Fig. 6).

## Conclusions

Here, we present 25 novel phage genomes from a novel lineage of large bacteriophages that we refer to as "moraphages". These phages were identified primarily in freshwater systems, indicating that aquatic environments are home to an abundance of novel large viruses. These novel phages have genes encoding many unique proteins that are seldom seen in other jumbo phages, most notably chaperones such as DnaJ and DnaK. They were also found to lack several genes for proteins that have been discovered recently in other large phages, such as those encoding tRNA synthetases and the machinery needed to form a phage nucleus, suggesting that these phages have an infection strategy that differs from those of other described jumbo phages. These results contribute to our growing understanding of large phages in the biosphere and underscore the importance of large viruses to global viral diversity.

## Data Availability

Supplementary data files, such as genome annotations, complete moraphage amino acid and nucleotide sequences, and high-resolution figures, can be found in the following Zenodo repository: Moraphage_Supplementary_Datasets_and_Figures (10.5281/zenodo.15586673).
